# Multi-scale variations and future projections of dry-wet conditions over the monsoon transitional zone in East Asia: A review

**DOI:** 10.1016/j.fmre.2024.01.023

**Published:** 2024-03-07

**Authors:** Wen Chen, Jinling Piao, Shangfeng Chen, Lin Wang, Wei Zhao, Zhikai Wang, Qiulin Wang

**Affiliations:** a Department of Atmospheric Sciences, Yunnan University, Kunming 650500, China; b Center for Monsoon System Research, Institute of Atmospheric Physics, Chinese Academy of Sciences, Beijing 100190, China; c CAS Key Laboratory of Regional Climate‑Environment for Temperate East Asia, Institute of Atmospheric Physics, Chinese Academy of Sciences, Beijing 100190, China; d College of Earth and Planetary Sciences, University of Chinese Academy of Sciences, Beijing 100049, China; e National Meteorological Center, China Meteorological Administration, Beijing 100010, China; f Hangzhou Meteorological Bureau, Hangzhou 310051, China

**Keywords:** East Asian monsoon transitional zone, Multi-scale variations, Future projection, Atmospheric teleconnection pattern, Atlantic multidecadal oscillation, Pacific decadal oscillation, Internal atmospheric variability, Global warming

## Abstract

The East Asian monsoon transitional zone (MTZ) is a northeast-southwest oriented belt between the wet monsoon areas and the northwestern dry areas of China with a fragile ecology and high climate sensitivity. The climate in the MTZ is characterized by strong instability and large variability, resulting in frequent occurrence of extreme weather and climate events. A number of studies have focused on the dry-wet characteristics from different perspectives, taking into account the increasing problems of water scarcity and ecological risks. This study reviews the multi-scale variations, underlying mechanisms and future projections of dry-wet conditions over the MTZ under global warming. The main findings over the last few decades are summarized as follows: 1) the interannual variability of summer precipitation is under the combined impacts of oceanic forcings and internal atmospheric teleconnection patterns at mid-high latitudes; 2) an interdecadal decrease in summer precipitation amount in the MTZ was observed in the late 1990s due to a Silk-Road pattern-like wave train triggered by the combined impacts of the Atlantic Multidecadal Oscillation-like SST warming over the North Atlantic and positive-to-negative phase shift of the Pacific Decadal Oscillation (PDO); 3) a pronounced drying trend has been observed during 1951–2005, which is mainly attributed to human activities and internal atmospheric variability, including increased aerosols, land-use changes, thermal forcing over the Tibetan Plateau, and the phase shift of the PDO; and 4) the summer precipitation in the MTZ is projected to increase under global warming with considerable uncertainties mainly due to internal atmospheric variability, including the Arctic Oscillation and the Polar-Eurasian pattern. This review attempts to provide a clear and systematic picture on the distinctive changing features of dry-wet conditions over the MTZ, and to attract the interest of the scientific community in climate change over this unique “transition” domain.

## 1 Introduction

**Fig. 1:**
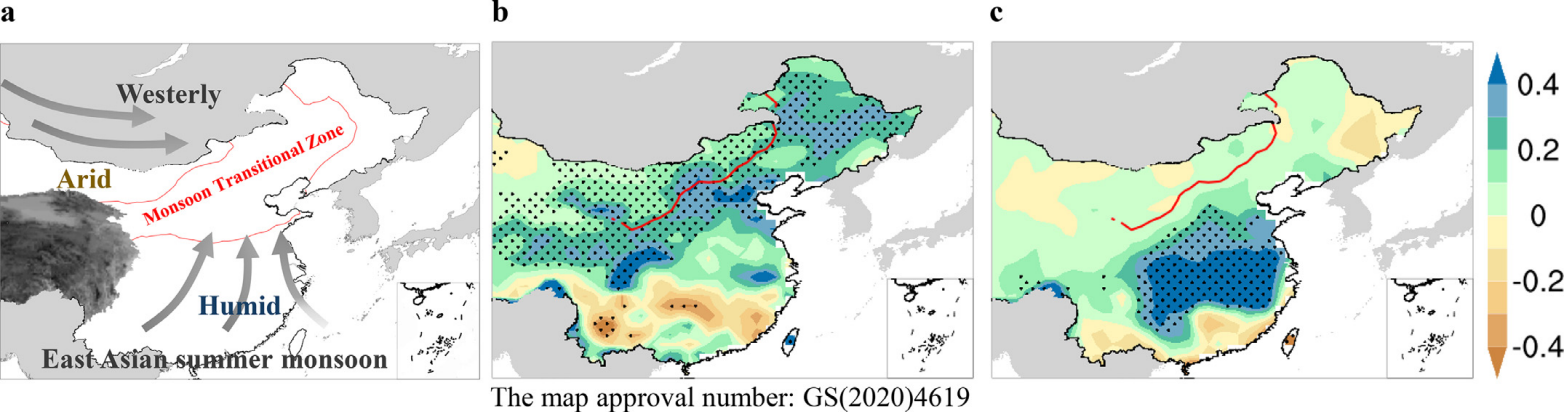
(a) Schematic of transition features of the MTZ domain. (b), (c) Summer (June–August) precipitation pattern (unit: mm d^−1^) regressed onto the EASM northern boundary index and the EASM intensity index during 1979–2022, respectively. The red lines in (b, c) represent the mean position of the EASM northern boundary. Dotted areas in (b, c) indicate regressed anomalies that are significant at the 95% confidence level. The northern boundary index and the EASM intensity index were calculated based on the definitions proposed by Chen et al. and Wang and Fan, respectively. The employed precipitation and atmospheric circulation dataset were derived from the NOAA's precipitation reconstruction data over land (Chen et al. 2002) and the National Centers for Environmental Prediction–National Center for Atmospheric Research (NCEP-NCAR) (Kalnay et al. 1996), respectively. Map of China is edited on Chinese standard map GS(2020)4619, which is the same as those in Figs. 2 and 4. (For interpretation of the references to color in this figure legend, the reader is referred to the web version of this article.)

The East Asian summer monsoon (EASM), an important component of the global monsoon system, exerts a dominant influence on climate over the Chinese mainland [1], [2], [3]. Extensive efforts have been made to study the seasonal march of the EASM system. The EASM usually begins over the South China Sea (SCS) in mid-May, coinciding with the onset of the rainy season over Southern China; it then moves northward to its northernmost position in late July, followed by the main rainbelt migrating to North China and Northeast China [4], [5], [6]. Any disturbance to the normal seasonal march of the EASM would have profound impacts on the advance/retreat of the main rainbelt, the length of the rainy season, and the dry-wet distribution over East China [[7], [8]]. In particular, the northernmost position that the EASM can reach―the northern boundary of the EASM, is subject to significant year to year variability, resulting in remarkably large precipitation variability over the area of its activity, which is widely referred to as the monsoon transitional zone (MTZ) (Fig. 1a) [[9], [10]]. Geographically, the MTZ is a northeast-southwest oriented belt that mainly covers semi-arid and semi-humid climate zones, including the eastern part of Inner Mongolia, Northern and Northeastern China. It is not only the transition between monsoon and non-monsoon regions, but also the transition from forest to steppe and desert steppe, which coincides well with the northern farming-pastoral zone of China [[11], [12]]. Due to its unique “transition” nature, the MTZ is featured by typical ecosystem vulnerability, showing high sensitivity to climate change compared to the adjacent monsoon regions and arid regions [[13], [14]]. Located at the interface between the EASM and the mid-latitude westerly circulation, the climate here shows extremely strong instability and large variability, especially dry-wet conditions, resulting in frequent occurrence of climate-related disasters [[15], [16]]. Since the 1990s, northern China has experienced a prolongation of consecutive dry-days and extreme summer droughts [17], posing a serious threat to the already existing water scarcity issues and ecological security. For example, a severe drought swept across seven provinces in northern China in the summer of 2014, which even became the most severe drought in the past 60 years [18].

Although the MTZ is adjacent to the conventional monsoon domain, it has quite distinctive features of the dry-wet variability. The main drivers include (1) the EASM system, (2) the mid-high latitude westerly circulation, and (3) local land surface processes [[15], [19], [20], [21]]. In contrast to the monsoon climate, which depends on the EASM intensity, the precipitation here shows a rather weak connection with the EASM intensity. Instead, it is directly influenced by the south-north fluctuations of the EASM northern boundary (Fig. 1b, c). In the past, it was generally accepted that the stronger the EASM becomes, the more northward the EASM boundary shifts, resulting in more precipitation amount over the MTZ. However, recent studies have suggested that this relationship does not always exist, leading to a relatively weak impact of the EASM intensity [22], [23], [24]. In terms of the influencing factor at mid-high latitudes, the westerly circulations play a dominant role in the dry-wet variability of the MTZ. On the one hand, wave-like teleconnection patterns propagating via the westerly can induce cyclonic/anticyclonic circulations over Northeast Asia, which then modulate the water vapor transport and local convection, leading to anomalous precipitation in the MTZ [[15], [20], [25], [26]]. On the other hand, anomalies in the intensity or position of the mid-latitude westerly jet can directly cause precipitation anomalies in the MTZ by modulating local meridional-vertical circulation [27]. In addition to the aforementioned large-scale atmospheric circulations, several studies have also highlighted the importance of local land surface processes [[12], [19], [28]]. As a transition between different terrestrial ecosystems, there are significant spatial-temporal changes in the land surface characteristics (e.g., vegetation cover, soil moisture), which have indispensable impacts on precipitation changes over the MTZ. Overall, the dry-wet conditions over this unique transitional zone contain large variations and complicated features under multiple forcing factors.

It is now widely recognized that the continued rising temperature driven by increasing greenhouse gas (GHG) emissions has induced widespread and profound impacts on human society and the Earth's ecosystems [[29], [30]], particularly in semi-arid regions with fragile ecosystems and high sensitivity to climate change [31]. Due to the unique “transition” nature mentioned above, the MTZ has been considered as one of these typical regions that are more vulnerable to global warming-induced climate changes. For instance, one of the strongest warming signals in recent decades is centered over the MTZ, which is more significant than those over the adjacent wet monsoon regions and arid regions in Northwestern China [32]. In this context, it has become an urgent issue to project future changes in the dry-wet conditions over the MTZ in response to global warming, considering the high dependence of rain-fed agriculture and ecosystems on precipitation. Both paleoclimate simulations and future projections suggest that the northern boundary of the EASM is expected to shift northwards under global warming, mainly due to the strengthening of the western Pacific subtropical high (WPSH) and the intensified land-sea thermal contrast [[33], [34]]. This further favors more moisture being transported northwards, leading to an increase in precipitation over the MTZ [35], [36], [37], [38]. Although the precipitation is projected to increase under global warming, there are significant uncertainties in the model simulations [39], which mainly arise from the external forcing (e.g., GHGs, aerosols, land use/land cover changes, and radiative forcing) together with the internal atmospheric variability [40], [41], [42]. It has been demonstrated that the internally-generated atmospheric circulation trend patterns similar to the Arctic Oscillation (AO) and the Polar-Eurasian (PEU) pattern dominate the uncertainties in the simulated precipitation trends over the MTZ. Despite the increasing attention paid to the future climate changes over the MTZ, previous studies have not been well organized to clearly illustrate the projected future dry-wet changes and the main influencing factors related to the external forcing and internal variability.

This paper reviews previous studies and attempts to provide a systematic picture of multi-scale variations and future projections of the dry-wet changes over the MTZ. Section 2 introduces various definitions of the MTZ domain based on different climate variables. Dry-wet changes on interannual and interdecadal timescales together with the long-term trend are summarized in Section 3, with main emphasis on the relative importance of the EASM system and the mid-high latitude westerly circulations. Section 4 mainly presents future projections of the dry-wet conditions under global warming and discusses uncertainties due to internal atmospheric variability. The summary of this study is given in Section 5.

## 2 Definitions of the monsoon transitional zone

The concept of the transitional climate zone was initiated by previous work [43], which is referred to as the boundary region between continental-scale climate zones. The region is typically characterized by strong gradients in climate variables and climate instability, making it highly vulnerable to natural disturbances and particularly anthropogenic global change. The MTZ—the transitional climate zone between the EASM domain and the arid regions in Northwestern China, consists mainly of semi-arid and semi-humid climate zones with annual total precipitation amount in the range of 200–400 mm. Numerous studies have defined the domain of the MTZ from various aspects, which can generally be classified into two categories based on the influential range of the EASM and climate types, respectively.

**Fig. 2:**
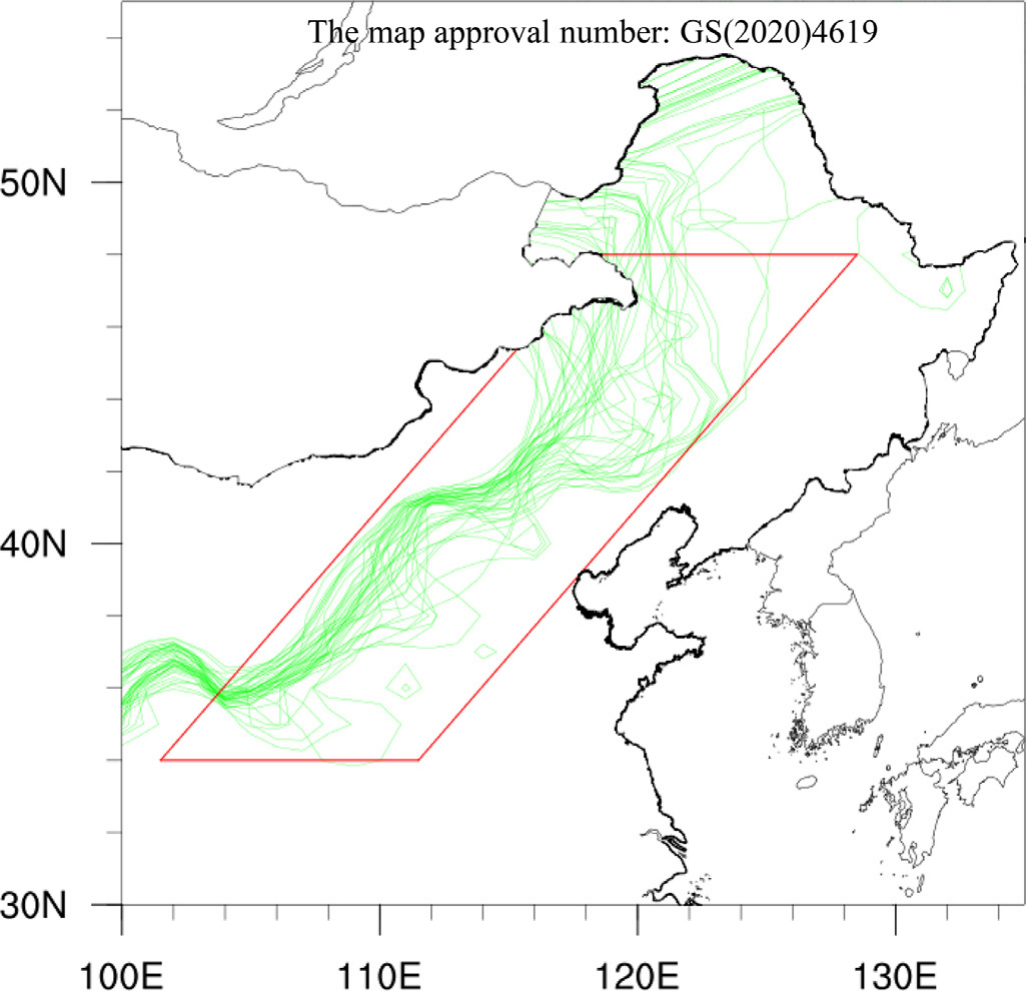
**2 mm day^−1^ isochrones of precipitation amount in boreal summer (i.e., MJJAS-averaged) with a 3-year running window (green lines)**. The green lines indicate the EASM northern boundaries based on the definition proposed by Chen et al. The red box represents the selected MTZ region. The employed data in this figure are 160 station records from the Chinese National Meteorological Center from 1979 to 2022.

The northern boundary of the EASM exhibits large interannual variability, leading to significant fluctuations and large gradient of climate conditions in its activity scope, which is defined as the MTZ (Fig. 2). For the northern boundary of the EASM, the proposed definitions can be divided into subtypes based on monsoon precipitation, air mass characteristics including moisture and temperature, and lower-tropospheric southwesterly winds over East Asia, respectively [10]. The former uses only one meteorological component, precipitation, making it the easiest and most straightforward. With the seasonal march of the EASM, the monsoon rainbelt begins with surges of rain over the SCS in mid-May, moves northward through North and Northeast China in mid-July, and finally reaches the northernmost location [4]. Based on the heavy convective precipitation during the onset of the SCS monsoon, Qian and Lee identified the northern boundary of the EASM as 4 mm/day isochrones of pentad mean precipitation [44]. To better study the EASM at longer timescales, Chen et al. suggested that 2 mm/day isoline of summer (May–September) precipitation can sufficiently depict the location of the northern boundary of the EASM and its variability, which has a clear physical significance as a climatic, ecological and geographical boundary [45]. Huang et al. subtracted the northern boundary of the EASM as the northern edge of the monsoon area, which is defined as the region where the local summer minus winter precipitation rate exceeds 2 mm/day, and the local summer precipitation exceeds 55% of annual precipitation [46]. In addition to precipitation, wind fields [47] and meteorological variables linked to moisture, such as specific humidity [48] and precipitable water [[49], [50]], are also frequently adopted to describe the northern boundary of the EASM, considering that it is the interface between the EASM and mid-latitude westerly where warm-humid air masses and cold-dry air masses meet.

These monsoon-based definitions only put emphasis on the impacts of the EASM, although other systems or mechanisms may also play a considerable role in the formation of the MTZ. For instance, Wang et al. showed that the mid-latitude westerlies, local evaporation, and the EASM each contributes about one-third of the water vapor input to summer precipitation over the MTZ, suggesting that the EASM is not the only influencing factor [51]. Furthermore, it is noted that impacts of evaporation on climate change cannot be ignored in the context of global warming, which is highly sensitive to changes in wind speed, temperature, radiation, and vegetation type. In this regard, several studies have defined the MTZ based on the Aridity Index (AI), which is calculated as the ratio of the annual sum of precipitation to the annual sum of potential evapotranspiration [[35], [52]]. Regions with AI smaller (larger) than unity are usually categorized as dry (wet) domains, since the precipitation amount is insufficient (sufficient) for the demands of evaporation. In essence, the AI represents the degree of water deficiency in a given region, and can be used to classify climatic types: Humid (AI > 0.65), Dry-subhumid (0.5 < AI < 0.65), Semi-arid (0.2 < AI < 0.5) and Arid (AI < 0.2) [[53], [54]].

So far, multiple studies have demonstrated that the AI can simply and reasonably indicate the terrestrial dryness/wetness over East Asia [[35], [55], [56]]. According to this, the MTZ can be defined as the domain with 0.2 < AI < 0.65 over East Asia, which is the “transitional zone” with a combination of semi-arid and dry subhumid environments [[35], [57]]. It is worth noting that although different definitions were proposed from different perspectives, they show high consistency in describing the general features of the MTZ domain [[21], [58]].

## 3 Multi-scale variations of dry-wet conditions over the MTZ

The dry-wet conditions over the MTZ are directly subject to the seasonal march of the EASM circulation, with more than half of the annual total precipitation amount observed in July and August when the EASM reaches its northern boundary [[15], [59]]. Different from the monsoon climate that is dominated by the EASM system, the summer precipitation here is under the combined impacts of multiple influencing factors at low and mid-high latitudes, and thus has rather complex changes [[15], [57]]. In view of this, a number of studies have been conducted on the features and underlying mechanisms of summer precipitation changes at interannual and interdecadal timescales together with its long-term trend [[16], [19], [26], [60]].

### 3.1 Interannual variability

The summer precipitation over the MTZ exhibits significant interannual variation, with a dominant period of 2–4 years [59]. Previous studies have suggested that the key influencing factors on the interannual timescale include not only local climate systems (e.g., the EASM and mid-latitude westerly circulation), but also remote forcings such as the North Atlantic Oscillation (NAO) and sea surface temperature (SST) anomalies in the Pacific and Atlantic [[20], [26], [61]]. These factors can together modulate atmospheric vertical motions and moisture supply, leading to complex interannual variation features of the dry-wet conditions over the MTZ.

**Fig. 3:**
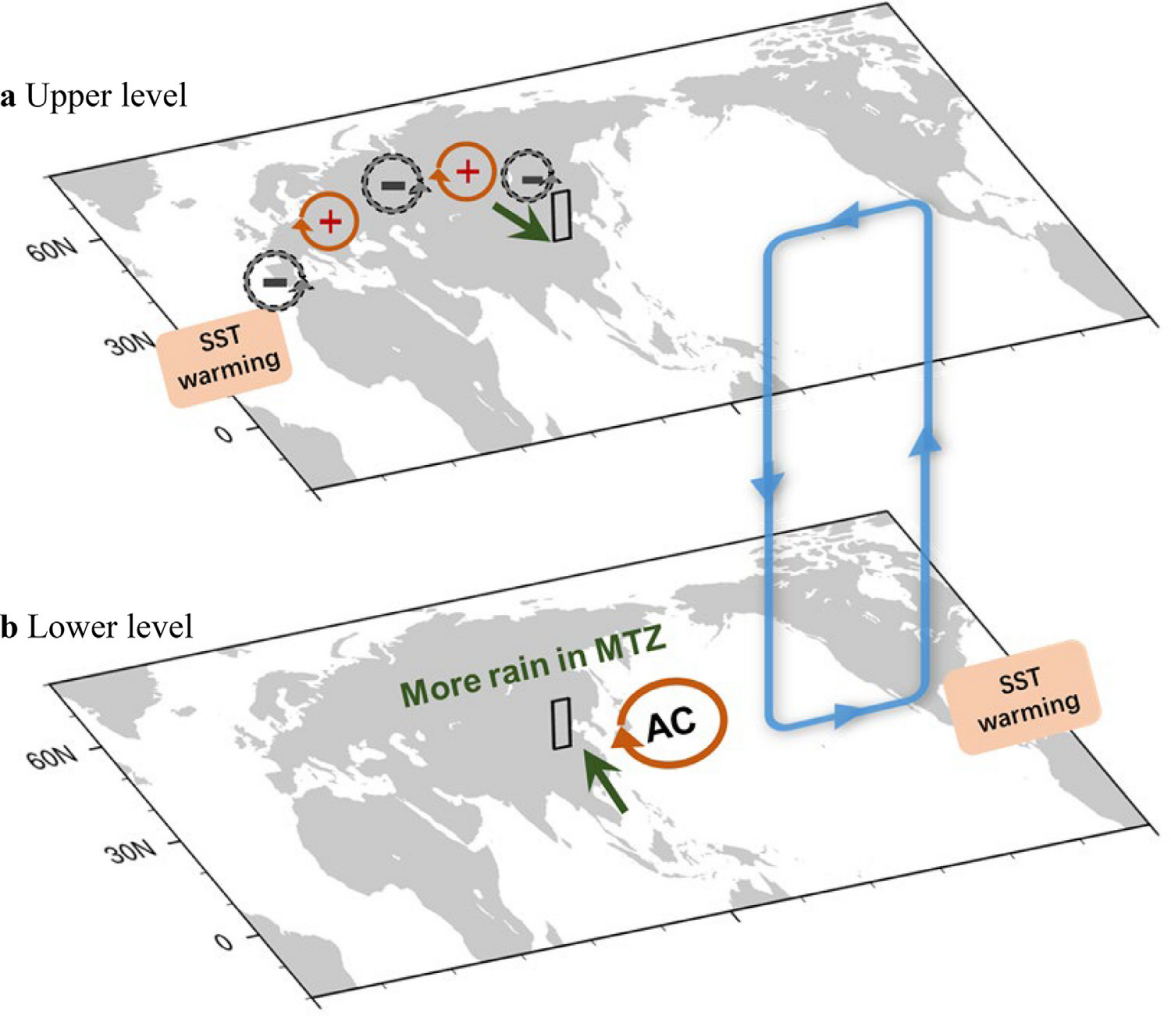
**Schematic of impacts of NTA SST anomalies on the MTZ precipitation variation in August over the MTZ via a mid-latitude atmospheric teleconnection over Eurasia (a) and a tropical pathway (b).** Red and gray circles in (a) represent positive and negative anomalies of 300 hPa geopotential height, respectively. Blue arrows in (b) indicate anomalous Walker circulation induced by NTA SST warming with ascending and descending branches in the NTA and the tropical central Pacific, respectively. Red circle over the WNP denotes the anticyclonic circulation in lower troposphere. Green arrows and black boxes in (a) and (b) indicate wind anomalies and the MTZ domain, respectively.

In recent years, an increasing number of studies have been focused on investigating the underlying mechanism for the interannual variation, emphasizing the crucial role of both the tropical and mid-high latitudes forcing. From a local perspective, the precipitation increase over the MTZ is always accompanied by an anomalous anticyclone over the tropical western North Pacific (WNP) and negative geopotential height anomalies over the MTZ. The latter is related to an atmospheric teleconnection pattern over the Eurasian continent, which shows a significant relationship with the interannual precipitation variation by inducing anomalous water vapor convergence and ascending motions [[59], [61], [62]]. Observations and sensitivity model experiments showed that both local signals are under the combined impacts from an El Niño-like SST warming in the tropical central-eastern tropical pacific (TCEP) during the preceding winter and SST warming in the northern tropical Atlantic (NTA). On the one hand, numerous studies have shown that the preceding winter El Niño events play an important role in the formation of the anomalous anticyclone over the WNP in the following summer via associated SST anomalies over the Indian Ocean and the NTA, and the local air-sea interaction over the WNP [[63], [64], [65], [66]]. On the other hand, in the absence of ENSO signals, the SST anomalies over the NTA still exert considerable impacts on precipitation anomalies via two pathways (Fig. 3). One pathway is in the tropics (Fig. 3b). The NTA SST warming causes an anomalous Walker circulation over the tropics, with ascending and descending branches in the tropical Atlantic and the tropical central Pacific, respectively. The latter branch can further induce the anomalous anticyclone over the WNP via a Gill-type atmospheric response [59]. The other pathway is in the mid-latitudes (Fig. 3a). The NTA SST warming can trigger an atmospheric wave train propagating from the North Atlantic to East Asia to induce the negative geopotential height anomalies over the MTZ, which contribute to precipitation increase by inducing local ascending anomalies [[20], [36], [62], [67], [68]].

In addition to oceanic forcing, several studies have also emphasized the important role of internal atmospheric processes in modulating dry-wet conditions over the MTZ, such as the PEU, the circumglobal teleconnection (CGT), the Scandinavian (SCA), and Eurasian teleconnection (EU) [[61], [69], [70], [71], [72]]. For instance, the PEU pattern is one of the dominant modes of geopotential height anomalies north of 15°N, whose positive (negative) phase is characterized by negative (positive) height anomalies over the polar region and positive (negative) ones over the northern China and Mongolia [73]. Previous studies suggested that the PEU pattern is significantly correlated with the summer precipitation over the MTZ, with its positive phase favorable for precipitation decrease via modulating the intensity of the Northern East Asian low, which can lead to anomalous water vapor flux divergence and descending motions [[71], [72], [74]]. Lin showed that the PEU, the SCA together with the CGT, exert a combined impact on rainfall anomalies over Northern and Northeastern China, with the two former patterns explaining more than 20% of the rainfall variance in North China [74]. Subsequently, Piao et al. (2018b) further found that the May NAO plays a considerable role in the summer PEU pattern, thus contributing to the interannual precipitation variation over the MTZ [61]. They also pointed out that the May NAO-MTZ summer precipitation connection is not stable, with weak signals before the late 1970s but a significant negative relationship afterwards. This enhanced relationship is suggested to be due to the strengthening of the May NAO connection with the following summer PEU pattern, which might be attributed to the inter-decadal contraction of the Northern Hemisphere circumpolar vortex.

**Fig. 4:**
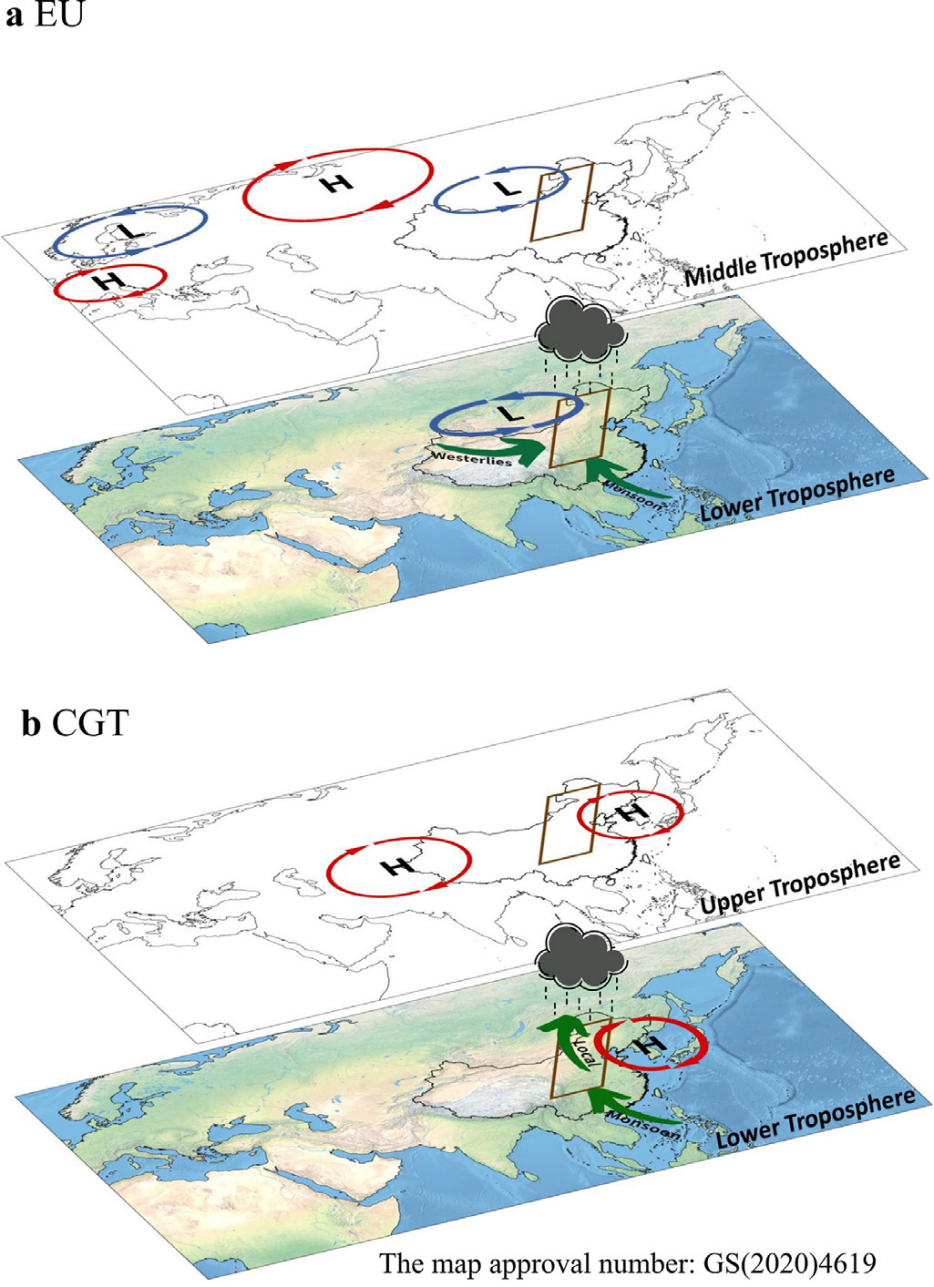
**Schematic of impacts of the EU teleconnection (a) and the CGT pattern (b) on interannual variability of summer precipitation in the MTZ.** Green arrows indicate increased water vapor supply from the corresponding system to the MTZ, and red circles represent positive circulation anomalies.

Due to the limited water resources, the interannual precipitation variation over the MTZ depends not only on the aforementioned dynamic factors associated with vertical motions, but also on thermodynamic effects related to moisture supply [[26], [51], [75]]. Based on the atmospheric water vapor balance equation, Piao et al. (2018a) found that the interannual variation of moisture supply over the MTZ is largely explained by the large-scale water vapor transport, but shows weak connections with the local land surface processes [75]. Considering that the MTZ is under the control of the mid-latitude westerly jet where transient eddies frequently occur, they further compared the relative importance of the mean flow and the transient eddies on the water vapor transport anomalies, and revealed the dominant role of the former factor, namely the stationary component. Subsequently, recent studies demonstrated that the EU and CGT patterns are the two critical modes governing the large-scale water vapor transport over the MTZ, both of which can significantly modulate the moisture input transported by the EASM and the mid-latitude westerlies (Fig. 4) [[26], [75]]. By examining the moisture input across four boundaries, observational results showed that the EASM-dominated meridional moisture transport is more important than the mid-latitude westerly-related zonal component. Wang et al. further utilized a Lagrangian particle dispersion model, the FLEXPART, to quantify the relative contribution of the main moisture sources [51]. Their results confirmed that the EASM system has a larger impact on the interannual variation of summer precipitation over the MTZ from a moisture supply perspective. In comparison, the interannual variation of moisture supply over East Asia is also subject to the EASM-dominated water vapor transport via the southern boundary [[76], [77]]. However, the water vapor transport is mainly linked to SST anomalies over the Tropical Indian Ocean and the South China sea [78], which poses a contrast to the dominant role of the mid-latitude atmospheric circulation patterns in the case for the MTZ.

### 3.2 **The interdecadal shift around the late-**1990s

Over the past decades, widespread warming has been observed over large areas of Northern Asia, with one of the particularly strong signals centered in the surrounding of the MTZ [[23], [60], [79], [80]]. In this context, an abrupt interdecadal decrease in summer precipitation is identified around the late 1990s, with the decreased amount accounting for nearly 15.7% of the climatological mean [[15], [60]]. Under the combined impacts of rising temperature and decreasing precipitation, northern China and Mongolia have experienced a prolonged and severe drought [[81], [82], [83]], which is highly unusual for the last 2000 years [[79], [84], [85]]. In particular, both the frequency and intensity of droughts are significantly increased in the MTZ since the late 1990s, which is more robust compared to changes in other regions across the China domain [86]. Due to its high ecological vulnerability, the MTZ showed a stronger response to climate changes compared to adjacent monsoon domains, posing a severe threat to the human society and ecological security [[83], [87]]. In accompany with the unusual drought development and human activities, the widely-distributed lakes over the Mongolian Plateau have encountered significant shrinkage in the past decades, which particularly enhanced since the late 1990s, with the decreasing rate of the number of lakes reaching nearly 34% in Inner Mongolia of China [83]. Meanwhile, the consecutive droughts during the growing season have also had a significant impact on the pasture production, with nearly 32% of the decline in productions in concert with drought events [87].

**Fig. 5:**
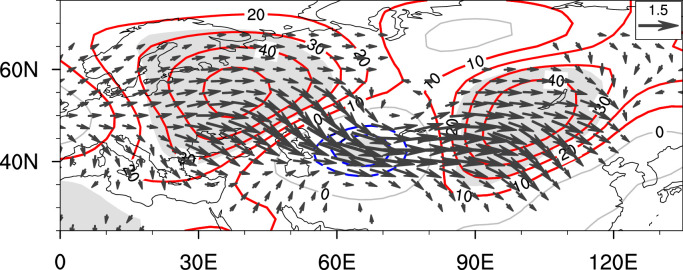
**Difference in geopotential height (contours; units: gpm) and wave activity flux (vectors; units: m^2^ s^−2^) at 200 hPa between 1999 and 2007 and the climatological mean state (1971–2000)**. Shading indicates the regions where the difference in geopotential height is significant at the 90% confidence level using the Student's *t-*test. Vectors less than 0.15 are omitted. The atmospheric circulation dataset is derived from NCEP-NCAR.

Given the serious socio-economic implications of this interdecadal change in the precipitation over the MTZ, a number of works have been conducted to investigate the key influencing factors and related physical mechanisms [[15], [25], [88], [89]]. The observations show that since the late 1990s, a wave-like teleconnection pattern with positive-negative-positive geopotential anomalies centered over Eastern Europe, Central Asia and Northeast Asia has emerged over the Eurasian continent, which is quite similar to the negative phase of the Silk-Road pattern (SRP) (Fig. 5) [[15], [25], [60]]. Among them, the positive center over Northeast Asia is suggested to cause anomalous descending motions [[15], [25], [27]] and moisture divergence mainly via modulating the meridional water vapor transport [[60], [90]], exerting dominant impacts on the interdecadal precipitation decrease over the MTZ. Compared to the thermodynamic effects related to water vapor anomalies, the dynamic effects connected with atmospheric circulation anomalies turn out to be the dominant factor for this interdecadal change [60]. In accompany, significant warming signals appeared over the North Pacific and Atlantic in summer SST differences between post- and pre-shift periods, which are suggested to be the main oceanic forcings for the above-mentioned atmospheric circulation anomalies [[15], [42]]. The warming over the North Atlantic bears much resemblance to the positive phase of the Atlantic Multidecadal Oscillation (AMO), which is the dominant mode of North Atlantic SST anomalies on the interdecadal time scale [91]; and the warming over the North Pacific shows close associations with the negative phase of the Pacific Decadal Oscillation (PDO) [92].

A few studies have highlighted the crucial impact of the AMO-like warming over the North Atlantic [[15], [25]]. For example, Wang et al. (2017b) suggested that although neither the AMO nor the PDO shows significant relationships with the SRP-like teleconnection on the interdecadal timescale, the positive phases of the spring and summer AMO are expected to significantly increase its frequency of occurrence based on the Monte Carlo bootstrapping resampling analysis [25]. Piao et al. further set up a series of sensitivity experiments and found that the AMO-like warming over the North Atlantic has a remarkable impact on the excitation of the SRP-like teleconnection, thus dominating the interdecadal decrease in the summer precipitation over the MTZ [15]. They further pointed out that the EASM has a limited contribution to this interdecadal change, showing no significant relationship with the summer precipitation over the MTZ on the interdecadal timescale [15]. In contrast, several studies have highlighted the significant role of the positive-to-negative phase shift of the PDO over the North Pacific, which is likely to favor warming over the Lake Baikal and weakening of the westerly jet through the air-sea interaction in the Pacific, thus contributing to the interdecadal changes in the summer precipitation by modulating the jet-related secondary meridional-vertical circulation [[27], [93]]. Several studies have discussed the combined effects of the PDO- and AMO-related circulation anomalies previously [[90], [94]]. Zhang et al. showed that when the PDO and AMO are out of phase, such as the situation after the late 1990s with warming over the North Pacific and North Atlantic, it favors the formation of the SRP-like teleconnection (negative phase) spanning across the Eurasian mid-latitudes, which bridges the AMO-related North Atlantic-European east-west mode with the PDO-related North Pacific barotropic atmospheric circulations, thus resulting in the interdecadal precipitation changes over East China [94]. Their combined impacts are further emphasized from the thermodynamic perspective [[60], [90]]. Piao et al. (2020a) showed that under the AMO- and PDO-related atmospheric teleconnection pattern, the moisture transported into the MTZ is significantly reduced due to the anomalous anticyclone over the MTZ, which remarkably reduces the water vapor amount transported from the Pacific and eastern Siberia, favoring the interdecadal precipitation change in the late 1990s [90]. Despite the above-mentioned close connection of the AMO and PDO with the interdecadal precipitation decrease over the MTZ in the late 1990s, it remains uncertain the underlying mechanism of how they force the concerned atmospheric circulation anomalies, which might be attributed to the insufficient understanding of the causes and mechanisms of the AMO and PDO.

### 3.3 Long-term trend

Over the past decades under global warming, North China has experienced the most significant drying trend compared to other regions in China, which is under the combined influence of rising temperature and decreasing precipitation [[86], [95], [96]]. Within this area, the MTZ shows stronger signals compared to the adjacent regions based on several drought indices (e.g., the AI, the standardized precipitation index and the standardized precipitation evapotranspiration index) [[35], [86]]. A previous work reviewed studies on the drought over East Asia and suggested that this drying trend together with the wetting trend in the Yangtze River valley, i.e. the widely mentioned “south flood-north drought”, was closely related to the interdecadal weakening of the EASM circulation [96]. The related anomalous northerly winds caused a reduction in the northward transport of water vapor, which further led to deficient rainfall amount in the MTZ. Some studies have paid attention to the role of internal atmospheric variability, such as the thermal forcing over the Tibetan Plateau, the warming over the Indian Ocean and western Pacific, and the phase transition of the PDO [[97], [98]]. For instance, it is suggested that the negative-to-positive phase shift of the PDO exerts remarkable impacts on the weakened EASM circulation by reducing the land-sea thermal contrast [[96], [99]] and exciting an anomalous Pacific-Japan/East Asin-Pacific-like teleconnection pattern [100]. In addition, the heating fields over the Tibetan Plateau in subsequent spring and summer experienced a significant weakening after the late 1970s, which is accompanied with the interdecadal SST warming in the western Pacific. The resulting land-sea thermal contrast further weakens the EASM, and thus contributes to the drying conditions in the MTZ [101].

**Fig. 6:**
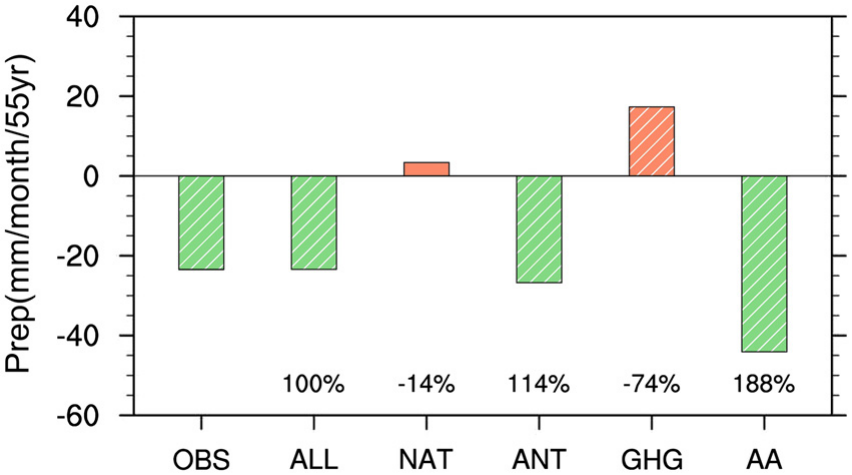
**Linear trends of precipitation (unit: mm month^−1^ 55 yr^−1^) in August averaged over the MTZ during 1951–2005 based on observational result (OBS) and multi-model ensemble simulations of historical run (ALL), natural forcing run (NAT), anthropogenic forcing run (ANT), GHG forcing run (GHG) and aerosol forcing run (AA) from CMIP5 models**. Details of the CMIP5 models can be seen in Table S1 in Zhao et al. The observational result is the average between precipitation trends that were calculated using the Climatic Research Unit TS 4.01 data (Harris et al. 2014) and the Global Precipitation Climatology Centre dataset (Schneider et al. 2017). Bars with slashes indicate linear trends exceed the 90% confidence level. The numbers at the bottom of the figure denote the relative contribution percentage of the corresponding external forcing.

In addition to the above-mentioned internal atmospheric variability, other studies also emphasized the substantial role of human activities on the drying trend, such as increased aerosols [[102], [103]] and land-use changes [[104], [105]]. Among these anthropogenic forcings, the dominant role of anthropogenic aerosol forcing has been increasingly proven in multiple numerical simulations in spite of the relatively large range of uncertainties (Fig. 6) [[106], [107]]. Currently, there are two different mechanisms by which aerosol increasement influences the drying trend of the MTZ. On the one hand, the anthropogenic aerosol forcing exerts direct radiative effects on the weakening of the EASM via reducing the land-sea thermal contrast. The resulting strong low-level northerly anomalies over eastern China suppress water vapor transport from the southern oceans, resulting in drier conditions over the MTZ [[16], [108], [109]]. On the other hand, increased aerosols have been suggested to increase the concentration of cloud droplet number and reduce the efficiency of raindrop collision through indirect radiative effect, leading to the decrease of local light rain frequency and rainfall amount [[110], [111], [112]]. In addition, different types of aerosols have distinct impacts on the wet/dry tendency of the MTZ [[102], [106]]. For example, the increase in sulfate aerosols enlarges the temperature gradient in the upper and middle troposphere, which causes northward movement of East Asian westerly jet and strengthens the EASM, and therefore favors the wetting trend over the MTZ [106]. However, opposite climate effects are detected in the case for black carbon aerosols, with decreasing precipitation induced in northern China [102]. In comparison with the anthropogenic aerosol forcing, the GHG forcing is expected to induce a wetting trend in the MTZ by enhancing the monsoon circulation over East Asia associated with the increased land-sea thermal contrast [[16], [108], [112]]. Last but not least, the land-use changes are also considered to exert considerable role in the drying trend via inducing decrease in precipitation amount and increase in temperature [104], especially after the 1990s [105].

## 4 Future projections

The ongoing global warming is progressing with an unprecedented speed at least during the last 22,000 years, which is mainly attributed to GHG emissions by human activities [113]. According to the latest released Intergovernmental Panel on Climate Change Sixth Assessment Report, this unprecedented global warming has already induced widespread and abrupt climate changes in the Earth's climate system. The climate change has led to more frequent and more intense weather and climate extremes across most land regions, such as heatwaves and heavy precipitation [29]. In particular, it is suggested that some climate changes have already passed the tipping point and may not be reversed for centuries to millennia, especially those in regional/global hydrological cycles, global sea level and ice sheets [[30], [114], [115]]. Due to the typical vulnerable ecological environment, semi-arid zones such as the MTZ are likely to be more prone to the global warming-induced disturbances [116]. Consistently, climate types with the annual total precipitation amount in the range of 250–300 mm have been shown to have the strongest response of surface evapotranspiration to global warming [117]. For instance, Mongolia has suffered from unprecedented compounding warm and dry anomalies in the past two decades, which are likely to have passed the tipping point [85]. Under this circumstance, it has gradually become a frontier and hot spot for projecting future changes in dry-wet conditions over the MTZ under global warming [[24], [34], [38]].

There is a growing consensus among previous studies on a remarkable increase in the projected precipitation amount over the MTZ [[23], [39], [118], [119]], which is accompanied by more frequent and more intense extreme climate events [[35], [120], [121]]. Several studies have used warm periods in the paleoclimate with atmospheric CO_2_ concentrations similar to the present level, and considered them as robust analogs for near-future scenarios [[33], [58], [122], [123]]. Both proxy records and paleoclimate model simulations showed that the strengthening of the land-sea thermal contrast during warm periods (e.g., the mid-Holocene, the mid-Pliocene) is expected to induce the intensification and westward shift of the western Pacific subtropical high (WPSH) [[33], [58]]. This favors the northward shift of the northern boundary of the EASM and the northward migration of the monsoon rainbelt, resulting in increased precipitation over the MTZ [122]. Recent studies show high consistency in projected future changes [[34], [35], [37]]. By using future projections from phase 5 of the Coupled Model Intercomparison Project (CMIP5), Wang et al. suggested that the northern boundary of the EASM is likely to shift northward and have stronger interannual variability, which may result in increased precipitation over the MTZ with more frequent occurrence of extreme events [35]. Subsequently, a recent study using CMIP6 model simulations showed that the strengthening of the land-sea thermal contrast together with the weakening of the East Asian subtropical westerly jet tends to enhance the EASM meridional circulation and shift the northern boundary of the EASM to the northwest, leading to increased precipitation over the MTZ via intensified moisture transport [34]. Piao et al. (2021c) further compared the future projections between CMIP5 and CMIP6 and indicated that the increased precipitation amount is mainly dominated by the intensified vertical moisture advection in both the two model ensembles, with stronger changes projected by the CMIP6 counterparts [37]. The main differences between the two model ensembles lie in the fact that the intensified vertical moisture advection is attributed to thermodynamic effects associated with humidity changes induced by global warming in CMIP5, but shows a remarkable dependence on dynamical effects associated with atmospheric circulation in CMIP6 (see Fig. 12 of Piao et al. [37]). They inferred that the stronger land-sea thermal contrast projected in CMIP6 could have significant impacts, favoring the northward shift of the EASM northern boundary and monsoon circulations mentioned above, and then highlighting the dynamic forcing on the increased precipitation in the MTZ. Similar studies have been performed over the adjacent Central Asia and East Asia, but both cases emphasized the thermodynamic effects on the projected precipitation changes, which accord well with the ‘dry-getting-drier’ mechanism [[124], [125]]. These discrepancies might lie in the higher sensitivity of the MTZ to climate changes, which results in stronger responses to the northward shift of the EASM northern boundary and monsoon circulations, thus underlining the dynamical effects compared to the surroundings.

Although a relatively high model consensus is recognized in the increased precipitation over the MTZ under global warming, there are still significant uncertainties that could substantially affect the regional climate assessment [[35], [38], [39], [42], [126]]. As is widely demonstrated in previous studies, uncertainties mainly arise from three sources: (1) diversities in model responses to the same climate forcing, (2) incomplete understanding of the future external forcing, and (3) processes intrinsic to the atmosphere, the ocean and their coupled system [[40], [127], [128]]. Among these, the role of the internal atmospheric variability has received special attention in recent decades [[41], [129], [130]], since the uncertainties associated with it belong to the spontaneous nature of the climate system itself, and are therefore irreducible and unpredictable. Focusing on the case of the MTZ, Wang et al. showed that uncertainties arising from climate models play a dominant role in the projected future dry-wet changes, which is in sharp contrast to the limited contribution form the scenario uncertainty [35]. Following this, Piao et al. further suggested that the internal climate variability superimposed on the external forcing dominates the uncertainties in the projected summer precipitation trends [42]. Here the external forcing mainly refers to the increasing emissions of GHGs related to human activities and radiative forcing associated with volcanic activity and solar output with a natural origin [131]. To distinguish between the internally-generated climate variability and externally-forced climate trend, large ensemble simulations run by a single model are considered an efficient tool and have been widely used to quantify the relative contributions of the two factors on the simulated climate changes [[41], [129], [132]]. Basically, each ensemble member shares the identical prescribed time-varying radiative forcing but is subject to a different set of initial conditions. Under this circumstance, the ensemble mean trends are mainly attributed to the external forcing, and the differences between the individual member simulations and the ensemble mean can represent the contribution of internal variability [[133], [134]]. Based on the model outputs of the 40 CCSM3 ensemble members, Piao et al. suggested that the external forcing favors the summer precipitation increase over the MTZ, which is consistent with paleoclimate model simulation results [42]. They further applied the EOF analysis in the “ensemble trend” domain during 2006–2060, and suggested that the dominant modes similar to the AO and PEU patterns exert combined impacts on the internally-generated uncertainties in the projected summer precipitation trend over the MTZ, which account for nearly 30% of the total variance.

## 5 Summary and discussion

In recent years, an increasing amount of research has focused on the dry-wet variability over the MTZ, since it is a special transitional zone with typical ecosystem vulnerability and high sensitivity to climate change. The relatively low amount of precipitation with strong variability frequently results in extreme weather and climate events, especially droughts, posing a growing threat to the already-existing water scarcity and ecological destruction issues. Under this circumstance, a number of studies have investigated dry-wet variability over the MTZ at different timescales, i.e., interannual, interdecadal changes and long-term trend, and projected future changes under global warming. Considering that the dominant influencing factors and related physical mechanisms vary among different timescales, this study provided a systematic literature review for a better understanding of the complex dry-wet variability over the MTZ.

On the interannual timescale, the summer precipitation over the MTZ is characterized by significant variations with a dominant period of 2–4 years under the combined impacts of oceanic forcings and internal atmospheric teleconnection patterns at mid-high latitudes. Oceanic forcings mainly include SST anomalies over the TCEP and NTA. On the one hand, an El Niño-like SST warming in the TCEP during the preceding winter favors an increase in precipitation over the MTZ via promoting the formation of the anomalous anticyclone over the WNP. On the other hand, the SST anomalies in the NTA, which are independent of ENSO signals, can also play an important role in the MTZ precipitation by not only exerting impacts on the formation of the anomalous anticyclone over the WNP, but also triggering an atmospheric wave train over Eurasia. In terms of internal atmospheric teleconnection patterns, the PEU, CGT, SCA and EU patterns together with the preceding May NAO have been found to be significantly related to precipitation anomalies over the MTZ via inducing anomalous vertical velocities and water vapor transport. Although previous studies have already evaluated CMIP6 models in simulating the climatological mean fields of dry-wet conditions over the MTZ and the northern boundary positions of the EASM [[21], [37]], there is a lack of related studies on the relationships between the main influencing factors and precipitation variations over the MTZ.

In addition to the interannual variation, the summer precipitation over the MTZ experienced an interdecadal decrease around the late 1990s, followed by a prolonged and severe drought afterwards. Observations and model sensitivity experiment results suggest that the AMO- and PDO-related atmospheric circulations play a crucial role in the precipitation decrease, both being responsible for anomalous descending motions and water vapor flux divergence. On the one hand, the AMO-like warming over the North Atlantic is suggested to trigger an SRP-like teleconnection pattern over the Eurasian continent with an anticyclonic center over the MTZ, and contribute to the decreased precipitation there. On the other hand, the positive-to-negative phase shift of the PDO favors a warming over the Lake Baikal and weakening of the westerly jet, and the related secondary meridional-vertical circulation further contributes to this interdecadal decrease. Although the importance of the AMO and PDO for the abrupt decrease in precipitation in the 1990s has been widely reported, previous studies have also found that the two climate indices are not significantly correlated with the precipitation over the MTZ on the interdecadal timescale [[15], [25]]. It remains unclear whether their insignificant relationships arise from the limitation of the relatively short study period, or the competition between different oceanic forcings [94].

As for the long-term trend, during the second half of the 20th century, a significant drying trend was observed over northern China, accompanied by rising temperature and decreasing precipitation, with stronger signals identified over the MTZ compared to the adjacent regions. This drying trend is widely attributed to the interdecadal weakening of the EASM circulation, which weakens the water vapor transported northwards and thus contributes to deficient rainfall amount. For the underlying mechanism, some studies emphasized the importance of internal atmospheric variability, most of which are suggested to exert considerable impacts mainly via modulating the atmospheric teleconnection pattern and land-sea thermal contrast. Others highlighted the significant impacts of human activities, such as increased aerosols and land-use changes. Among them, anthropogenic aerosol forcing has been paid special attention, which can lead to the drying trend over the MTZ by reducing the land-sea thermal contrast and the efficiency of raindrop collision through indirect radiative effect. So far, the relative importance of the natural and anthropogenic forcings has remained controversial, which is partly attributed to the limitation of employed data length and model ensembles.

Regarding the future projections, the summer precipitation over the MTZ is expected to increase with the northward migration of the EASM northern boundary. They are mainly attributed to the intensification and westward shift of the WPSH induced by the strengthened land-sea thermal contrast under global warming. Moreover, CMIP5 and CMIP6 model ensembles further suggested that the projected increase in summer precipitation is largely due to strengthened vertical moisture advection, which is mainly attributed to thermodynamic forcing related to rising temperature in CMIP5 but dynamical processes associated with atmospheric circulation changes in CMIP6. This discrepancy might result from a stronger land-sea thermal contrast simulated by CMIP6 due to higher climate sensitivity, which leads to the further northward shift of the EASM northern boundary and accompanied monsoon circulations. Although the projected precipitation increase received relatively high model consensus, considerable uncertainties still exist among model simulations. Internal climate variability superimposed on the external forcing is thought to play a dominant role, with the dominant modes similar to the AO- and PEU-related patterns. In order to obtain more reliable and convincing future projections, further research is needed to reduce the uncertainties produced by the raw model simulations, which can not only help better understand future dry-wet changes over the MTZ, but also provide a valuable scientific basis for strategic decisions on mitigation and adaptation to potential climate change.

## Declaration of competing interest

The authors declare that they have no conflicts of interest in this work.
